# An Innovative Computational Strategy to Optimize Different Furnish Compositions of Tissue Materials Using Micro/Nanofibrillated Cellulose and Biopolymer as Additives

**DOI:** 10.3390/polym13152397

**Published:** 2021-07-21

**Authors:** Flávia P. Morais, Ana M. M. S. Carta, Maria E. Amaral, Joana M. R. Curto

**Affiliations:** 1 Fiber Materials and Environmental Technologies Research Unit (FibEnTech-UBI), University of Beira Interior, Rua Marquês d’Ávila e Bolama, 6201-001 Covilhã, Portugal; mecca@ubi.pt; 2 Forest and Paper Research Institute (RAIZ), R. José Estevão, 3800-783 Eixo, Portugal; ana.carta@thenavigatorcompany.com; 3 Chemical Process Engineering and Forest Products Research Centre (CIEPQPF), University of Coimbra, R. Sílvio Lima, Polo II, 3004-531 Coimbra, Portugal

**Keywords:** absorbency, commercial biopolymer additive (CBA), micro/nanofibrillated cellulose (CMF), softness, strength, tissue paper materials

## Abstract

The furnish management of tissue materials is fundamental to obtain maximum quality products with a minimum cost. The key fiber properties and fiber modification process steps have a significant influence on the structural and functional properties of tissue paper. In this work, two types of additives, a commercial biopolymer additive (CBA) that replaces the traditional cationic starch and micro/nanofibrillated cellulose (CMF), were investigated. Different formulations were prepared containing eucalyptus fibers and softwood fibers treated mechanically and enzymatically and both pulps with these two additives incorporated independently and simultaneously with drainage in the tissue process range. The use of these additives to reduce the percentage of softwood fibers on tissue furnish formulations was investigated. The results indicated that a maximum of tensile strength was obtained with a combination of both additives at the expense of softness and water absorbency. With a reduction of softwood fibers, the incorporation of additives increased the tensile strength and water absorbency with a slight decrease in HF softness compared with a typical industrial furnish. Additionally, a tissue computational simulator was also used to predict the influence of these additives on the final end-use properties. Both additives proved to be a suitable alternative to reduce softwood fibers in the production of tissue products, enhancing softness, strength and absorption properties.

## 1. Introduction

In recent years, there has been great growth potential in the tissue paper market. Factors such as the abundance of fibrous raw materials, the high demand for hygiene and health products and the evolution of socio-economic living standards have promoted this global growth. In addition, advances in manufacturing technologies for these materials have made the market increasingly competitive [1]. Tissue paper materials are increasingly used in consumer goods products such as napkins, facial tissues, paper towels, toilet papers, diapers and tissue masks. Tissue papers are produced to meet the desired properties for a specific application in the market such as a low basis weight, softness, strength and absorption [2]. To optimize the process, a great deal of effort been made to improve the properties of the raw materials used in the production of these papers [1,2,3,4]. The highest quality tissue products are produced with a blend of hardwood and softwood fiber pulps. The production of tissue papers with 100% eucalyptus fibers generally presents a higher softness and absorption; however, the strength is degraded during the creping and converting processes in the tissue manufacture [3,4]. Softwood fibers are introduced because these pulps provide strength to the papers and ensure the paper machine’s runnability [5,6,7,8]. The fibers can also be subject to modification processes such as enzymatic and mechanical modifications. Refining is an important process in the development of functional tissue properties; however, it is associated with a large energy consumption [9,10]. The application of enzymes enhances not only the development of the tissue properties but also the reduction of energy costs associated with the refining process [11,12].

In addition to the modification of fiber pulps, chemical additives are used to modify properties or improve the production process for different materials. Different chemical additives used in the tissue paper industry are designed and applied according to different needs such as dry strength agents, wet strength agents, softeners and lotions [13,14]. Usually, these additives are cationic as the fibers carry negative charges and they are also more effective in much lower concentrations than anionic polymers. Nevertheless, these cationic additives normally adhere in excess to the paper, negatively affecting the creping process and the product quality [14]. The addition of strength agents can ensure that tissue products remain integrated when applied in wet conditions while the addition of debonding agents can improve the softness and bulk of these products [15,16,17,18,19,20,21]. However, reaching a balance between these two properties is a challenge because the conditions that maximize the softness are those that minimize strength properties. This balance can be achieved by selecting fibers, refining treatments and adding chemical polymers. Therefore, the tissue manufacturer must balance these factors in terms of cost and effect. The tissue paper industry provides the design, development and production of many chemical and polymeric additives [22].

The application of innovative and versatile additives such as biopolymers and micro/nanofibrillated cellulose (CMF), replacing synthetic additives currently used, is a sustainable strategy to be considered in order to produce high-quality tissue premium products with innovative features. Biopolymers as additives can be new natural and green approaches to new possibilities and advances in modifying or improving the final end-use tissue properties [23]. The same is true for CMF as an additive that has been extensively investigated for its application in tissue products due to its high specific surface area, excellent mechanical properties, flexibility, biocompatibility, biodegradability and lack of toxicity, among other properties [24,25,26,27]. The furnish formation is also affected by the CMF incorporation as an additive. CMF decreases porosity and air permeability when added to the tissue structures. CMF content and structural changes present a proportional behavior. These decreases are caused by the CMF bonding with the cellulose fibers in the 3D network structures, closing the porous structure. Furthermore, this reduction in porosity is also correlated with the increase in the structure’s apparent density [28].

The presence of CMF can present an impact on the structure porosity because of the increasing number of hydrogen bonds present in this nanocellulose. The increase of these bonds is related to its increase in the surface/volume ratio due to the nanosized scale [28]. By filling the large pores created by the 3D fiber network, the CMF incorporation contributes to smaller pore dimensions and a narrower distribution of the structures, providing abundant pores with a uniform size and, consequently, a decrease in the properties of water absorption capacity and capillarity penetration [24,25]. The flexibility of CMF can be a filler for large pores in structures [25]. Although the mechanical properties of tissue products are improving, CMF also brings a difficulty to the dewatering of papermaking furnishes. The fibrillation degree influences the dewatering capacity of pulp suspensions. The drainage time increases depending on the CMF fibrillation degree. Particles resulting from fibrillation are more easily incorporated into the fiber network, partially closing the pores between the fibers and consequently limiting the wet network’s ability to drain water. This drainage difficulty is also caused by the increased water retention capacity of CMF. However, this dewatering of pulp suspensions containing CMF can be improved with the use of retention aids and also with cationic starch-treated CMF with colloidal silica [28]. In addition to a negative impact on dewatering, the accumulation of CMF particles in the closed circuit of the tissue machine can increase the viscosity of the recirculating water, thus affecting processability. Therefore, there is a need to study the feasibility of using CMF to reduce the fiber content. Our study [24] confirmed that the use of 1 to 8% of CMF incorporated into tissue paper formulations, along with a reduction of hardwood and softwood fiber content, achieves a trade-off between the final end-use tissue properties and fiber blend costs, with drainability between 26 and 32 °SR (Schopper–Riegler degree). In this range of drainability, the furnish formulations are able to produce high-quality tissue papers without difficulties in tissue paper machine runnability and efficiency [2,24].

The application of these additives leads to the optimization of strength properties without compromising the softness and absorbency properties of these types of papers. These strategies can allow a reduction of softwood fibers in the production of tissue paper without affecting the strength performance and decrease the overall tissue manufacturing cost [24,27,28,29,30]. The aim of this work was to perform a comparative study between different tissue furnish compositions and the incorporation of two additives, a commercial tissue biopolymer additive (CBA) as a cationic starch substitute and CMF, and their effect on the properties of the final end-use tissue paper. For this purpose, the fiber and suspension drainability characterizations of different investigated samples were performed. Laboratory isotropic structures with a light basis weight of 20 g/m^2^ were prepared and the softness, tensile strength and absorbency properties were evaluated.

## 2. Materials and Methods

### 2.1. Materials

**Figure 1:**
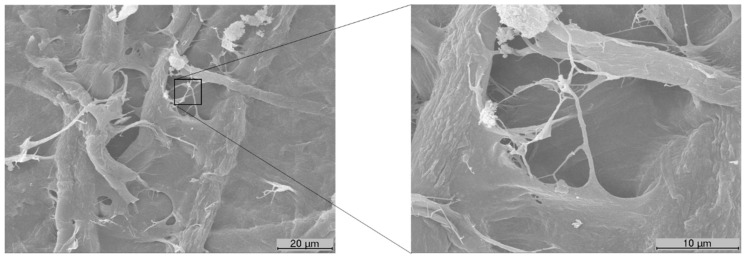
SEM image of the CMF additive, highlighting a high magnification image (3000×) where it is possible to verify the micro and nanofibrils of this sample.

An industrial, never-dried, bleached eucalyptus kraft pulp and an industrial, air-dried, bleached pinus kraft pulp were used in this work after disintegration according to ISO 5263-1. The additives used in this study were CBA and CMF. This commercial tissue additive, CBA, is a cationic polymeric solution specially designed as a replacement for cationic starch. CBA presents a viscosity of 500–900 cP, a pH of 2.1–4.0 and a density of 1.030 ± 0.040 g/cm^3^ at 20 °C (data supplied by a commercial enterprise). CMF was supplied by a research institute and obtained by mechanical (refining and homogenization) and enzymatic (cellulase) treatments using an industrial bleached eucalyptus kraft pulp. This sample was characterized in our previous work, which can be used for more detailed information [24]. CMF (Figure 1) presents an intrinsic viscosity of 1577 mL/g (ISO 5351), a carboxyl group content of 8.4 ± 0.2 mmol/100 g (TAPPI T 237 om-93), a suspension drainability degree superior to 80 °SR (Schopper–Riegler) and 56% of particles below 1000 nm, representing 10% of particles below 200 nm [24].

### 2.2. Enzymatic and Mechanical Treatments

The softwood pulp was beaten in a PFI mill at 4000 revolutions under a refining intensity of 3.33 N/mm (ISO 5264-2) followed by an enzymatic treatment. The enzymatic treatment was performed according to the method described by Morais et al. [12]. The treatment was carried out at a consistency of 4% at pH 7 at 40 °C with continuous mechanical agitation to ensure an efficient mass transfer for 60 min and 30 mg of enzyme/g of pulp.

### 2.3. Tissue Formulations

Table 1 presents the various tissue formulations (investigated samples) carried out in this study. The different formulations were performed to study the effect of additives in the tissue structure properties and understand their implication in the reduction of softwood pulp usually used in this type of paper. The first formulation presents a similar combination of eucalyptus fibers and softwood fibers typically used in tissue mills. All of the samples presented similar drainability properties around 25 °SR, which is recommended for tissue paper products and means that drainage and processability are in the range for the tissue process and, consequently, processability is not negatively impacted [2].

### 2.4. Preparation of Tissue Structures

The tissue structures were produced in a batch laboratory sheet former according to the method described by Morais et al. [6,7]. An adaptation to ISO 5269-1 was used, namely, the suppression of the pressing operation and the production of structures with a basis weight of 20 g/m^2^. The tissue structures were prepared with the different formulations described in Table 1. Finally, all samples were conditioned at 23 ± 1 °C, with a relative humidity of 50 ± 2%, according to ISO 187.

### 2.5. Characterization of the Morphological, Drainability and Chemical Properties

The morphological properties of pulp fibers and formulations were evaluated automatically by an image analysis using a fiber analyzer, the MorFi equipment (TECHPAP, Grenoble, France).

In order to characterize the surfaces of the different formulations, the obtained tissue structures were coated with gold using a Sputter Quorum Q 15 OR ES (Laughton, East Sussex, UK) to be analyzed by scanning electron microscopy (SEM) using a Hitachi S-2700 model (Tokyo, Japan) with an accelerating voltage of 20 kV at different magnifications. ImageJ, an image analysis software, was also used to characterize the pore properties of the samples. The SEM images were processed and analyzed using a methodology with defined criteria for the stabilization of the average of the measured values [31].

The chemical functional groups were analyzed using Fourier transform infrared spectroscopy with attenuated total reflectance (FTIR-ATR) at a wavelength of 600–4000 cm^−1^ with a resolution of 4 cm^−1^.

The drainability of pulp suspensions and formulations was evaluated by the °SR method (ISO 5267/1). Triplicate assays were performed for both methods.

### 2.6. Characterization of the Tissue Properties

#### 2.6.1. Structural Properties

The structures produced were characterized in terms of basis weight (ISO 12625-6), thickness and bulk (ISO 12525-3). The basis weight was obtained by the quotient between the average mass of each structure and the respective area (0.02138 m^2^). The thickness was obtained using a Frank-PTI micrometer. The bulk was obtained by the quotient between the thickness and the basis weight of the structures. Additionally, the porosity (apparent theoretical porosity) of the structure was also calculated according to the following equation:

(1) $$Porosity\;\left(\%\right)=100\times \left(1-\frac{\rho_{structures}}{\rho_{cellulose}}\right)$$

where *ρ_cellulose_* was the density of the cellulose (1.5 g/cm^3^) [12] and *ρ_structure_* was the apparent density of the structures (g/cm^3^).

#### 2.6.2. Softness Properties

Softness was assessed using a Tissue Softness Analyzer, the TSA (Emtec). A two-step measurement was used. The first was a noise measurement where the real softness (TS7) was measured. The second step was a deformation measurement where stiffness, plasticity, elasticity and hysteresis were measured. The handfeel (HF) parameter value was calculated using both steps.

#### 2.6.3. Strength Properties

Strength properties were determined according to ISO 12625-4 using the tensile index and calculated according to the following equation:

(2) Tensile Index Nm/g=breakWG

where *break* was the breaking force (N), *W* was the sample width (50 mm) and *G* was the sample grammage or basis weight (g/m^2^).

#### 2.6.4. Absorbency Properties

The absorbency properties were determined by the methods of water absorbency capacity per unit of mass and Klemm capillary rise.

The immersion absorption method was used to determine the water absorption capacity per unit of mass of the samples. The assay was carried out according to an adaptation of ISO 12625-8. The sample was immersed for 30 s. At the end of that time, the sample was removed from the water and left to run on a support that guaranteed an amplitude of 30° for approximately 1 min. Finally, the amount of water that the sample was able to absorb during this time was determined. The assay was performed in triplicate for each sample.

The Klemm capillary rise method was measured through an adaptation of ISO 8787. The assay consisted of the water rising in the sample (50 mm wide) at a certain time. The capillary rise was recorded at 10, 20, 30, 60, 180, 300 and 600 s. Four readings were performed for each sample. All of the assays were performed in a controlled temperature and humidity laboratory.

### 2.7. Computational Studies

SimTissue, a tissue simulator, was developed specifically for furnish management and optimization by FibEnTech-UBI members, in the scope of Project InPaCTus. This tissue simulator was used to compare different end-use final tissue properties and to access them for the different formulations to those performed experimentally [24].

A 3D fiber-based computational simulator was used to model the tissue structures as planar random networks. This simulator is open-source software, and the code is available on GitHub (https://github.com/eduardotrincaoconceicao/voxelfiber, accessed on April 2021). A more detailed description of this 3D simulator can be found in Conceição et al. and Curto et al. [32,33]. The fibers are represented by a voxel chain and modeled according to fiber dimensions and properties such as the fiber length and width ratio, flexibility, fiber wall thickness and lumen dimensions [34]. The structural properties were obtained following this computational study and the resulting 3D computational structures. The computational studies were performed using MATLAB^®^ (R 2020a, 9.8.0.1323502, MathWorks, Natick, MA, USA).

## 3. Results and Discussion

### 3.1. Characterization of Pulp Fibers and Tissue Formulations

**Figure 2:**
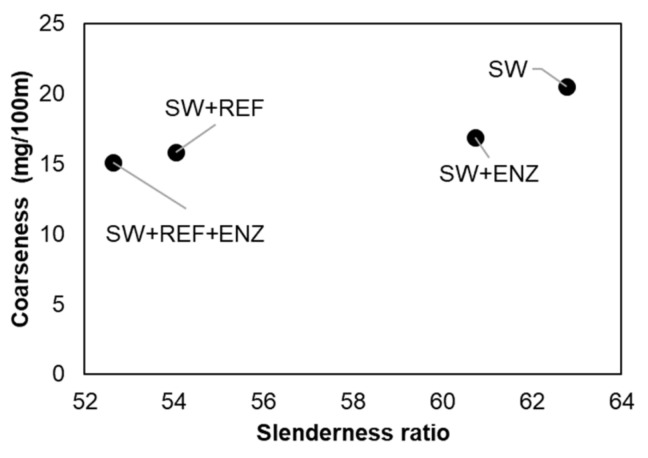
Coarseness as a function of the slenderness ratio of softwood fiber pulp (SW) with enzymatic treatment (SW + ENZ), with refining (SW + REF) and with the combination of both fiber modification treatments (SW + REF + ENZ).

In a first approach, the characterization of the raw materials used in the study was carried out. The eucalyptus pulp presented a length weighted by length of 0.729 ± 0.003 mm, a width of 19.1 ± 0.0 µm, a coarseness of 6.31 ± 0.04 mg/100 m and fine elements of 38.5 ± 0.4% in length. The softwood pulp presented a length weighted by length of 1.889 ± 0.052 mm, a width of 30.1 ± 0.2 µm, a coarseness of 20.52 ± 3.23 mg/100 m and fine elements of 36.6 ± 4.2% in length. In this study, only softwood pulp was subjected to fiber modification treatments, namely, refining and enzymatic treatments, as this eucalyptus pulp is already an industrial pulp suitable for the production of tissue papers. These treatments were carried out in order to improve fiber flexibility and inter-fiber bond properties with a greater strength three-dimensional matrix [35]. Analyzing the treatments separately, the refining effect was more pronounced than the enzymatic treatment. The results indicated that refining decreased the length weighted by length by 8% and coarseness by 30% and increased the fiber width by 7% and the fine content by 17% while the enzymatic treatment decreased the fiber length weighted by length by 2% and the coarseness by 18% and increased the fiber width by 23% and the fine content by 4%. The combination of these two treatments decreased the fiber length weighted by length by 10% and the coarseness by 26% and increased the fiber width by 7% and the fine content by 11% compared with the untreated pulp. Figure 2 presents an analysis of coarseness as a function of the length/width ratio (slenderness ratio) for the softwood samples. The results indicated that two groups can be considered with the fiber modification treatments applied. In the first one, the softwood fibers without treatment (63 of length/width ratio and 20.52 mg/100 m of coarseness) and with enzymatic treatment (61 of length/width ratio and 16.92 mg/100 m of coarseness) presented a higher slenderness ratio and higher coarseness. In the second group, the refined softwood fibers (54 of length/width ratio and 15.81 mg/100 m of coarseness) and with both treatments (52 of length/width ratio and 15.09 mg/100 m of coarseness) presented a lower slenderness ratio and lower coarseness. This suggests that the first group was more susceptible to presenting better tissue properties of softness and absorbency than the second group, which was more susceptible to presenting higher strength properties [7]. Additionally, the first group presented °SR between 12–14 and the second group presented 55–66 °SR. The mechanical refining and the combination of both treatments promoted a more efficient fiber fibrillation and consequently decreased the suspension drainability.

**Figure 3:**
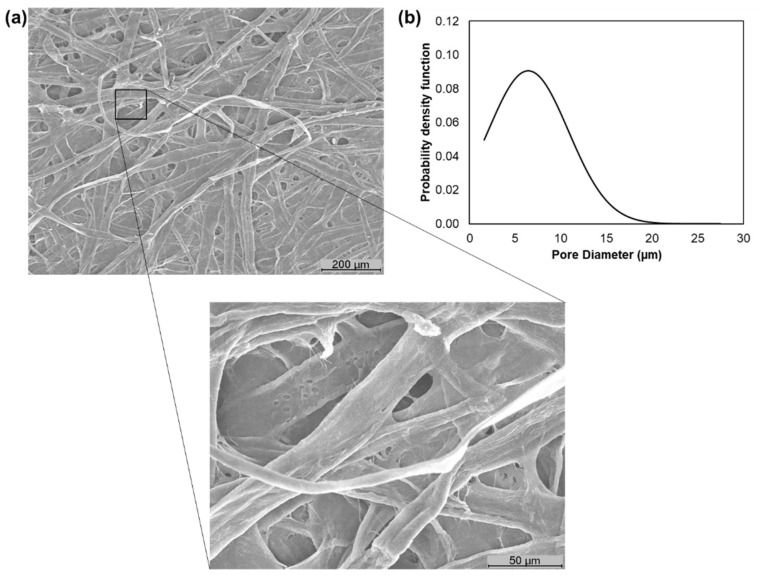
SEM image of the softwood pulp with refining and enzymatic treatment with a high magnification image (500×) (**a**) and its pore diameter distribution (**b**).

The softwood pulp with the refining and enzymatic treatment was also assessed through SEM images (Figure 3a). This treatment made it possible to modify the properties of the softwood pulp, promoting inter-fiber bonds and the combination of key structural properties such as the pore dimension and distribution. The pore properties depend on the fiber properties and the mechanical and enzymatic processes to which they are subjected [36]. The results indicated that these treatments had an impact on the fiber dimension and flexibility, resulting in a structure with a surface porosity of 83 ± 1% and a pore diameter distribution of 35% between 2 and 10 µm and 65% between 10 and 27 µm (Figure 3b).

**Figure 4:**
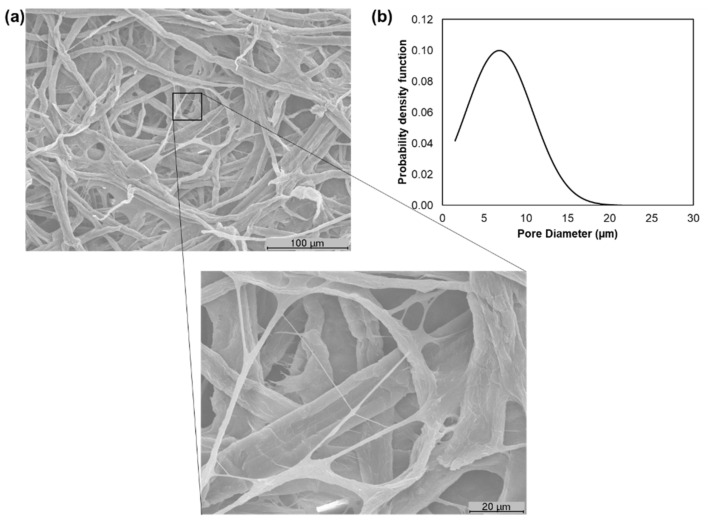
SEM image of tissue formulation 4 (75% eucalyptus pulp + 25% softwood pulp-treated + 2% CBA + 2% CMF) with a high magnification image (1000×) where is possible to verify the inter-fiber bonding between CMF and the structure in more detail (**a**) and its pore diameter distribution (**b**).

In a second approach, the characterization of the tissue formulations was carried out. As previously reported, this study compares two versatile additives: CBA and CMF. The MorFi analyzer is only able to evaluate the fiber suspension properties so the presence of the CBA does not affect its fibrous composition. The formulations with 75% eucalyptus pulp and 25% softwood pulp-treated and with incorporated additives presented a length weighted by length between 0.717 and 0.729 mm, a width between 19.4 and 19.9 µm, coarseness between 7.78 and 8.27 mg/100 m and fine elements between 32.7 and 35.7% in length. With a reduction of the softwood fibers, the formulations with 90% eucalyptus pulp and 10% softwood pulp-treated and with incorporated additives presented a length weighted by length between 0.719 and 0.722 mm, a width between 19.1 and 20.1 µm, coarseness between 6.77 and 7.61 mg/100 m and fine elements between 30.4 and 32.8% in length. Additionally, these formulations presented an °SR degree between 20 and 25 °SR. Although the softwood pulp presented low drainability, its combination with eucalyptus pulp and with different additives allowed the tissue formulations to present a range of °SRs suitable of producing premium tissue materials [2] without compromising the processability. Figure 4 presents the SEM image of the formulation 4 (75% eucalyptus pulp + 25% softwood pulp-treated + 2% CBA + 2% CMF) as well as its pore diameter distribution. The 3D structure matrix resulting from this formulation was presented as a multi-structured material with bonding between CMF fibrils and pulp fibers. The CBA addition also promoted a more closed structure with a surface porosity of 88 ± 1% and a pore diameter distribution of 40% between 1 and 10 µm and 60% between 10 and 22 µm (Figure 4b).

**Figure 5:**
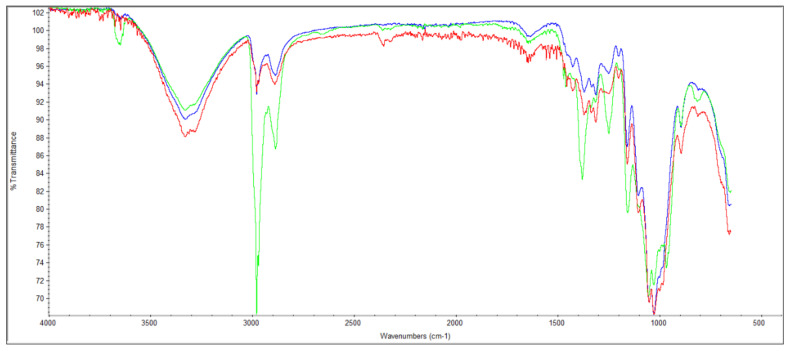
FTIR-ATR spectrum of formulation 1 (blue), formulation 2 (red) and formulation 3 (green).

To complement this study, the FTIR-ATR technique allowed us to chemically characterize, identify and quantify the formulations with CBA and CMF, evaluating the existence of physical retentions in the structure without new chemical bonds between these additives and the cellulose eucalyptus and softwood fiber 3D matrix. Figure 5 shows the FTIR spectrum of formulation 1 (a typical furnish mixture at a tissue mill), formulation 2 (with CBA incorporation) and formulation 3 (with CMF incorporation). The typical absorption bands of hydroxyl groups between 3000 and 3500 cm^−1^ indicative of the –OH stretching of the intra- and inter-molecular interactions of hydrogen bonds were verified for the three formulations. A –OH band with a higher area was observed for the structures with CBA. The biggest differences in the FTIR spectrum were found in the bands between 2800 and 3000 cm^−1^. This range corresponds with the =C–H stretching in the methyl groups of cellulose and hemicellulose. The band with the highest intensity in this range was observed for the formulation with CMF followed by the formulation with CBA and formulation 1. Additionally, the bands between 2200 and 2400 cm^−1^ correspond with the –OH asymmetrical stretching vibration of the carboxylic acid due to the enzymatic treatment applied to the materials [12]. The bands between 1500 to 1700 cm^−1^ were due to the C=O stretch of hemicelluloses. Other characteristics of cellulose were also observed between 800 and 1500 cm^−1^ such as the angular deformation of C-H and primary alcohol C-O bonds, the absorption band of C–O–C bonds, the *β*-glycosidic bonds between glucose units, C=H stretching, C=O stretch vibration in the syringyl ring and carboxylate ion group vibration.

### 3.2. Characterization of the Properties of the Final End-Use Tissue

#### 3.2.1. Structural Properties

**Figure 6:**
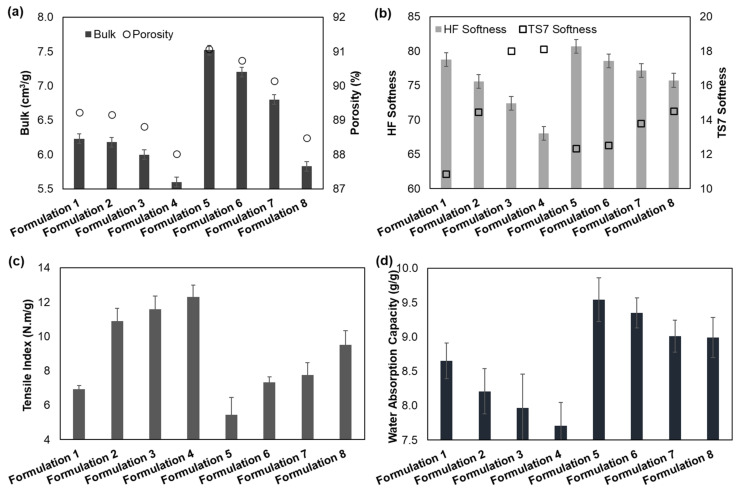
Properties of bulk and porosity (**a**), softness (**b**), tensile index (**c**) and water absorption capacity per unit of gram (**d**) of all tissue formulations.

**Figure 7:**
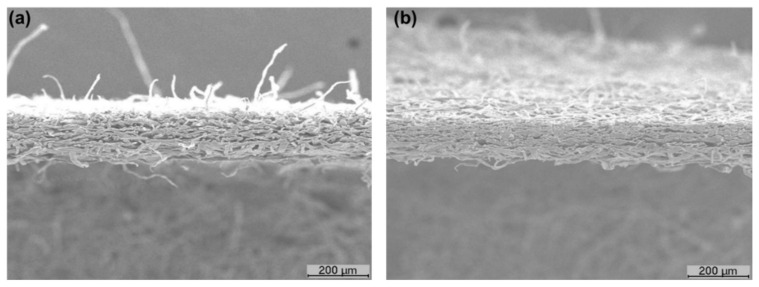
SEM images of the cross-section of the tissue structures prepared with formulation 1 (**a**) and formulation 4 (**b**).

**Figure 8:**
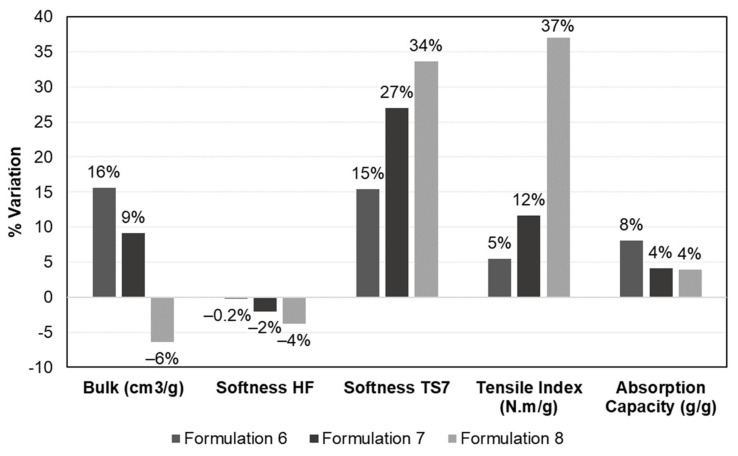
Percentage of variation in the properties of bulk, softness HF, softness TS7, tensile index and water absorption capacity of formulations 6, 7 and 8 compared with formulation 1 obtained with SimTissue.

Bulk and porosity are essential parameters for the quality of tissue products that influence the softness and liquid absorption properties [6,7]. Figure 6a shows, for the different tissue formulations, the behavior of the structural properties. The addition of CBA in formulation 2 promoted a decrease in bulk and porosity by 1% and 0.1%, respectively, compared with formulation 1. The addition of CMF (formulation 3) showed a tissue structure with lower bulk (<4%) and porosity (<0.5%) compared with formulation 1. The combination of these versatile additives (formulation 4) also promoted an increase of hydrogen and inter-fiber bonds, inducing a decrease of 11% in bulk and 1% in porosity of the tissue structures in comparison with formulation 1. This densification effect can be observed in the cross-section of the structures, as shown in Figure 7. With the reduction of the reinforcement fibers by 15% in the formulations, bulk and porosity were increased. This reduction promoted an increase of 20% in bulk and 2% in porosity when comparing formulations 1 and 5. The same trend in formulations 1–4 was observed in formulations 5–8; i.e., the combination of both additives presented a higher influence on the structural properties followed by CMF and CBA. Formulations 6, 7 and 8 promoted a reduction in bulk by 4, 10 and 22%, respectively, and a reduction in porosity by 0.4, 1 and 3%, respectively, compared with the control (formulation 5). The addition of both CBA and CMF in formulations with a softwood fiber reduction (90% eucalyptus fiber and 10% softwood fiber) allowed the obtaining of structures with increased structural properties compared with typical industrial structures (75% of eucalyptus fiber and 25% softwood fiber). The results indicated that formulations 6 and 7 increased the structure bulk by 16% and 9%, respectively, compared with formulation 1 (Figure 8). However, the combination of these two additives promoted a 6% reduction in bulk compared with this formulation typically used industrially.

#### 3.2.2. Softness Properties

Softness is also a characteristic that is highly appreciated by consumers, resulting in the combination of structure flexibility, bulk and surface softness. Softness properties of the different formulations are shown in Figure 6b. The HF (handfeel) values decreased with the additives loaded in the paper 3D matrix. The increase in fiber flexibility, fine elements and, consequently, the number of inter-fiber bonds promoted a more closed and compact 3D matrix with less bulk, resulting in a structure with less softness. The addition of CBA in formulation 2 promoted a decrease in softness HF by 4% compared with formulation 1. The addition of CMF (formulation 3) showed a tissue structure with lower softness compared with formulation 1 with a decrease of 8%. The combination of these versatile additives (formulation 4) also promoted a decrease of 14% in the softness HF of the tissue structure compared with formulation 1. The reduction of the reinforcement fibers in the formulations increased softness properties by 2% compared with formulations 1 and 5. As with bulk properties, the same trend was also observed for softness properties. Formulations 6, 7 and 8 promoted a reduction in softness by 3%, 4% and 6%, respectively, compared with formulation 5. Regarding Figure 8, the softness HF values decreased by 0.2%, 2% and 4% with the incorporation of CBA, CMF and the combination of both, respectively. The production of structures with a low content of SW fibers and versatile additives promoted a small reduction in softness, improving strength properties, as reported below, contributing to the formation of a fibrous structure with less stiffness [37].

Softness TS7 is inversely related to softness HF values; therefore, it is influenced by the presence of fibers in the structure surface. TS7 values increased with the addition of additives in the structure (Figure 6b). This result indicated that the fibers in the structure presented higher bonds, causing structure densification and rigidity. The fibers in the z-direction were more adherent to the paper structure, reducing the number of free fibers on the tissue surface compared with the structures without additives (Figure 7). Compared with formulation 1, softness TS7 of the tissue structure increased by 33%, 65% and 67% with the incorporation of CBA, CMF and both, respectively (formulations 2–4). With the reduction of the reinforcement fibers in the formulations, this property also increased by 13% compared with formulations 1 and 5. Additionally, formulations 6, 7 and 8 promoted an increase in softness TS7 by 2%, 12% and 18%, respectively, compared with formulation 5. Compared with formulation 1 (75:25), softness TS7 increased by 15%, 27% and 34% with the incorporation of CBA, CMF and both, respectively, in formulations with a softwood fiber reduction (90:10) (Figure 8).

#### 3.2.3. Strength Properties

The tensile index is an essential parameter not only for the product quality but also to ensure the paper machine runnability, which depends on the fiber intrinsic strength and the inter-fiber bonds. The results of the tensile index for each tissue formulation are shown in Figure 6c. The incorporation of additives promoted an increase in the tensile index of the tissue structures. Differences in strength improvements varied with the performance of CBA and CMF and became more noticeable with the combination of these additives. Formulation 3 with the addition of CMF showed improvements in the tensile index (+67% compared with formulation 1) related to formulation 2 with the addition of CBA (+57% compared with formulation 1). Formulation 4, with the incorporation of both additives, presented higher structure strength improvements by 77% compared with formulation 1. This result was corroborated by the results of the literature as the addition of CMF and chemical additives in the tissue structures improve strength properties of these products [13,25,27]. These additives promoted an increase in the surface area of the tissue structures due to the micro/nanofibrils and consequently formed stronger and more stable structures through inter-fiber interaction and hydrogen bonding [27]. This fact can also be verified by the densification of the tissue structures with the incorporation of both additives (Figure 7b).

Reinforcement fibers play a fundamental role in strength properties; therefore, the reduction of these fibers in formulation 5 promoted a 21% reduction of the tensile index compared with formulation 1. Additionally, formulations 6, 7 and 8 promoted an increase in the tensile index by 34%, 42% and 74%, respectively, compared with formulation 5. It is important to highlight that the strength gains obtained with the addition of CMF and CBA and the softwood reduction in the tissue structures were higher when compared with formulation 1. Despite the structures presenting a low basis weight and no pressing, these additives favored strength properties of these tissue structures. Additionally, as shown in Figure 8, the tensile index increased by 5%, 12% and 37% with the incorporation of CBA, CMF and both, respectively.

The commercial biopolymer additive used in this work was a cationic polymeric solution that is especially intended to be a substitute for cationic starch. Generally, cationic starch is used in the printing and writing paper industry with functions of a dry strength additive, emulsification of sizing agents, retention and drainage. This starch improves the formation, internal bonding, surface strength, tensile strength, writing and printing surface and energy consumption [38]. However, in the tissue paper industry, this starch has several inconveniences as the increase in strength properties promotes an abrupt reduction in softness properties. A solution found in the tissue industry was to design a practically constant biopolymer that would increase strength properties while preserving softness properties with a reduction of softwood fiber content. Furthermore, this additive allows the reduction of the contaminating organic load and costs as a result of reducing the use of cationic starch [38]. Therefore, the results of this work indicated that, compared with formulation 1, a typical industrial mixture can produce tissue structures containing 2% of the CBA and 10% of softwood fibers with 5% tensile index increases while maintaining the softness (Figure 8). This cationic polymeric solution also improved the inter-fiber bonding and CMF particle retention. This retention was proven by the 38% increase in strength properties with both additives (formulation 8) compared with formulation 1. The incorporation of CMF alone (formulation 7) only increased strength properties by 12% compared with formulation 1 (Figure 8).

**Figure 9:**
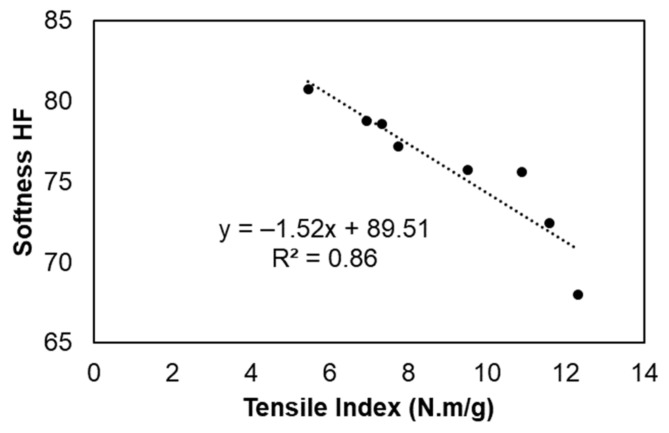
Correlation between the tensile index and softness HF of tissue formulations.

In addition, an inverse relationship between the properties of softness and strength can also be observed in these formulations (Figure 9); i.e., strength improvements present a negative impact on the tissue structure softness. The formulations promoted an increase of 1.52 units of softness HF for each unit with a decreased tensile index (y = −1.52x + 89.51; R^2^ = 0.89). In our previous studies, this relationship was also observed in formulations with only eucalyptus fibers and without additives in which the softness HF increased by 2.4 units per unit of the decreased tensile index [6,7]. This can contribute to the reduction of costs associated with the use of softwood fibers in industrial furnishes as there is a balance between the properties of softness and strength in the tissue structures with the use of these versatile additives.

#### 3.2.4. Absorbency Properties

**Figure 10:**
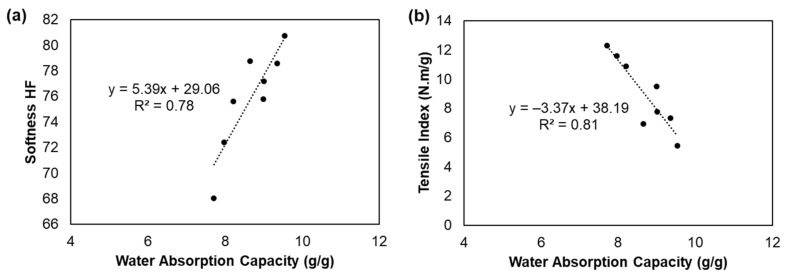
Correlation between the water absorption capacity and softness HF (**a**) and tensile index (**b**) of the tissue formulations.

Absorbency properties are essential in tissue products, mainly in paper towels and napkin s, depending on their fibrous structure. Tissue products with a more porous 3D matrix will present better water absorption as empty spaces for water interaction are more available [12]. Figure 6d presents the water absorption capacity of the tissue formulations in the study. Overall, the incorporation of CBA and CMF into the tissue formulations decreased the water absorption properties compared with the control. This result was also in line with the decrease in bulk and porosity properties (Figure 6a). This effect was observed from the SEM images in Figure 4a and Figure 7b in which the formulations with these additives presented a less porous surface with a more compact cross-section. The addition of CBA and CMF (formulations 2 and 3) presented a decrease of 5% and 8% of this property, respectively, compared with formulation 1. The incorporation of both additives presented higher absorption decreases by 11% compared with formulation 1. The reduction of the reinforcement fibers in the formulations also increased the absorption properties by 10% compared with the formulations 1 and 5. Additionally, formulations 6, 7 and 8 promoted a reduction in absorption by 2%, 6% and 6%, respectively, compared with formulation 5. Compared with formulation 1, absorption increased by 8%, 4% and 4% with the incorporation of CBA, CMF and both, respectively, in formulations with a softwood fiber reduction (90:10) (Figure 8). In this work, we also found a good linear correlation (R^2^ = 0.78) between the water absorption properties and the softness of the tissue structures made from these formulations (Figure 10a). The formulations promoted an increase of 5.39 units of softness HF for each unit of increased water absorption capacity (y = −5.39x + 29.06). The same inverse trend observed for the softness and strength properties was also verified for the absorbency and strength properties (Figure 10b). The tissue formulations promoted an increase of 3.37 units of water absorption capacity for each unit of decreased tensile index (y = −3.37x + 38.19; R^2^ = 0.81).

**Figure 11:**
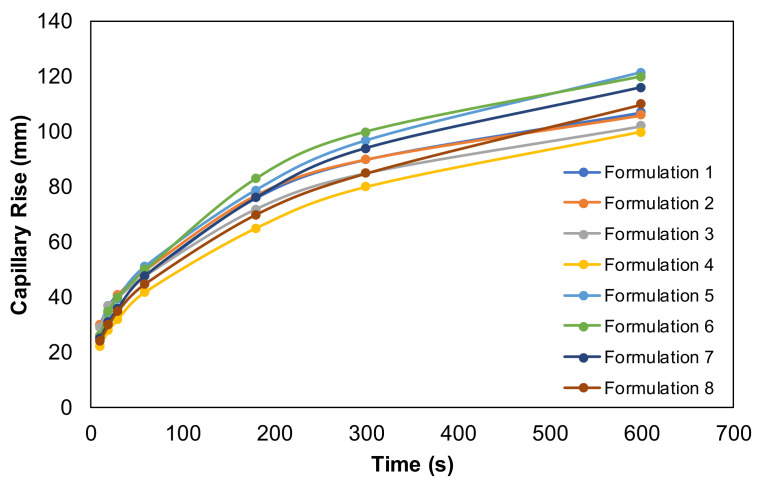
Klemm capillary rise method as a function of time for all tissue formulations.

The capillary rise of the structures depends on the adhesion and cohesion forces of the water molecules that ascend through the fiber walls or between the pores. Therefore, there must be a balance between the pore dimensions to promote the capillary rise efficiency. Inter-molecular bonds between fibers and water molecules in structures with higher pores can impair the capillary rise. On the other hand, in the case of channels that are too narrow, the water progression will be strangled by the limited space for capillary rise [39]. Figure 11 presents the Klemm capillary rise of the tissue formulations in this study. The same trend in the water absorption properties was observed in the capillary rise of tissue formulations. The formulations with a content of fewer softwood fibers presented better capillary ascents and the additives promoted the capillary rise decrease. The structures with a higher bulk and a more porous 3D matrix promoted a higher capillary rise. Comparatively of both additives, CBA promoted a better water affinity compared with CMF, corresponding with water capillary rise improvements.

These results indicated that a compromise could be found between the properties of softness, strength and absorbency with the incorporation of versatile additives in tissue formulations, reducing the incorporation of softwood fibers in an industrial furnish. This effect could promote a reduction in costs associated with the use of these fibers and also the mechanical and enzymatic refining applied to softwood fibers as the incorporation of these additives promoted structure strength improvements while maintaining the softness.

### 3.3. Computational Prediction of the CBA and CMF Performance in Formulations with 100% Eucalyptus Fibers

**Figure 12:**
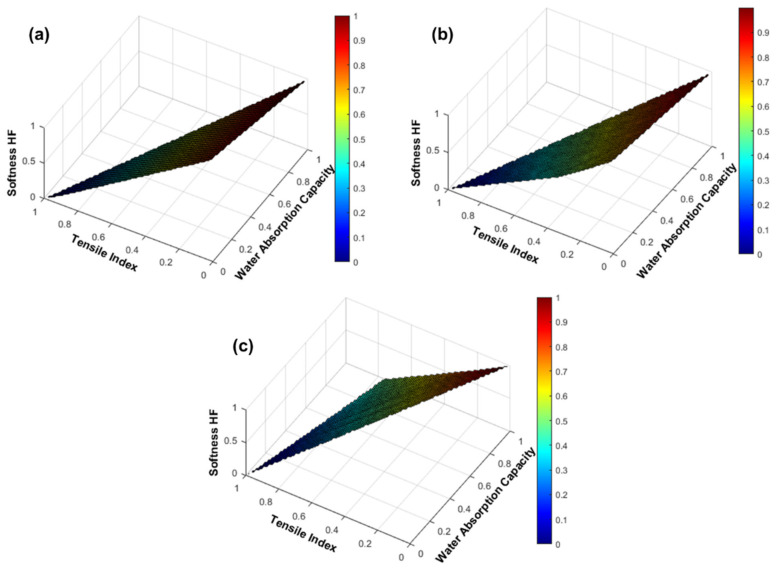
Evolution of the end-use tissue properties of softness HF, tensile index and water absorption capacity with the incorporation of CBA (**a**), CMF (**b**) and softwood fibers (**c**) in formulations with 100% eucalyptus fibers. All variables were normalized to present the same scale range.

The high manufacturing costs associated with tissue paper materials are due to the market softwood pulp prices and the energy consumption associated with the fiber modification processes such as refining or beating. Finding strategies to reduce these processes is one of the focuses of the research on the management and optimization of the furnish and each type of tissue product. For this reason, we developed a tissue simulator, SimTissue, with the ability to predict the final end-use properties of different hypothetical scenarios of industrial interest. Our approach in this study was to estimate improvements in tissue properties with the incorporation of CBA and CMF, separately, in formulations with 100% eucalyptus fibers compared with formulations containing eucalyptus fibers and softwood fibers (Figure 12). Note that these simulations were carried out with a reinforcing fiber with high strength properties, according to a set of pulps studied previously [6,7]. It is important to present the simulations of these fiber mixtures as they represent the limits achievable in the tissue properties.

As expected, the incorporation of CBA, CMF and softwood fibers promoted an improvement in strength properties with adverse effects on softness and absorbency. According to the tissue simulator, a formulation with 90% eucalyptus fibers and 10% softwood fibers promoted the production of a tissue product with a 68 softness HF, 15 Nm/g tensile index and 8 g/g water absorption capacity [24]. In order to replace this softwood fiber, the incorporation of 3% CBA allowed us to obtain improved tissue properties in the same range (72 softness HF, 15 Nm/g tensile index and 8 g/g water absorption capacity). The same trend was also verified for the incorporation of 14% CMF, presenting 70 softness HF, 15 Nm/g tensile index and 7 g/g water absorption capacity. However, in a previous work, we found that formulations with more than 10% CMF incorporation are not economically viable. The trade-off between the cost and the effectiveness of the formulations was achieved with the incorporation of CMF between 1 to 8%, presenting optimized properties at an industrial range [24].

This approach saves the consumption of softwood fibers in tissue production and is aimed at the use of biocompatible and biodegradable additives in the industrial production process. The simulator’s predictive capacity allowed the optimization and development of innovative formulations, saving laboratory and industrial resources.

## 4. Conclusions

This work presented experimental and computational approaches to evaluate and predict the effects of the incorporation of CBA and CMF with eucalyptus and softwood fibers on the softness, strength and absorbency properties of tissue paper materials. Overall, the addition of both additives enhanced tensile strength properties at the expense of reduced softness and absorbency properties, followed by CMF and CBA. This cationic polymeric solution also improved the inter-fiber bonding and CMF particle retention. With the softwood fiber reduction in the formulations, the incorporation of additives promoted a strength and absorbency increase with a low softness decrease. It was possible to obtain a trade-off between the tissue end-use properties with CBA and CMF additions separately. With this work, it was possible to produce tissue furnish formulations using these additives with a reduction of the softwood fiber percentage in a drainage range of the tissue industrial process. This suggested that these additives presented a good potential to be introduced in the production of high-quality tissue materials such as tissue masks or premium napkins and facial papers. These biocompatible and biodegradable additives can promote the maximization of eucalyptus fibers in industrial tissue production, optimizing the process and associated manufacturing costs.

## Figures and Tables

**Table 1:** Tissue formulations used in the study.

	Eucalyptus Pulp	Softwood Pulp with Mechanical and Enzymatic Treatment	CBA (Commercial Biopolymer Additive)	CMF (Micro/Nanofibrillated Cellulose)
Formulation 1	75%	25%	-	-
Formulation 2	75%	25%	2%	-
Formulation 3	75%	25%	-	2%
Formulation 4	75%	25%	2%	2%
Formulation 5	90%	10%	-	-
Formulation 6	90%	10%	2%	-
Formulation 7	90%	10%	-	2%
Formulation 8	90%	10%	2%	2%

## Data Availability

The raw/processed data required to reproduce these findings cannot be shared at this time due to legal or ethical reasons.
